# Supplementary material for: Identification of Tomato microRNAs in Late Response to Trichoderma atroviride
Source: Int J Mol Sci. 2024 Jan 28;25(3):1617. doi: 10.3390/ijms25031617 (PMC10855890; doi:10.3390/ijms25031617)
Supplement: Supplementary file 1 [file ijms-25-01617-s001.zip › New Figure S1_Olmo.pdf]

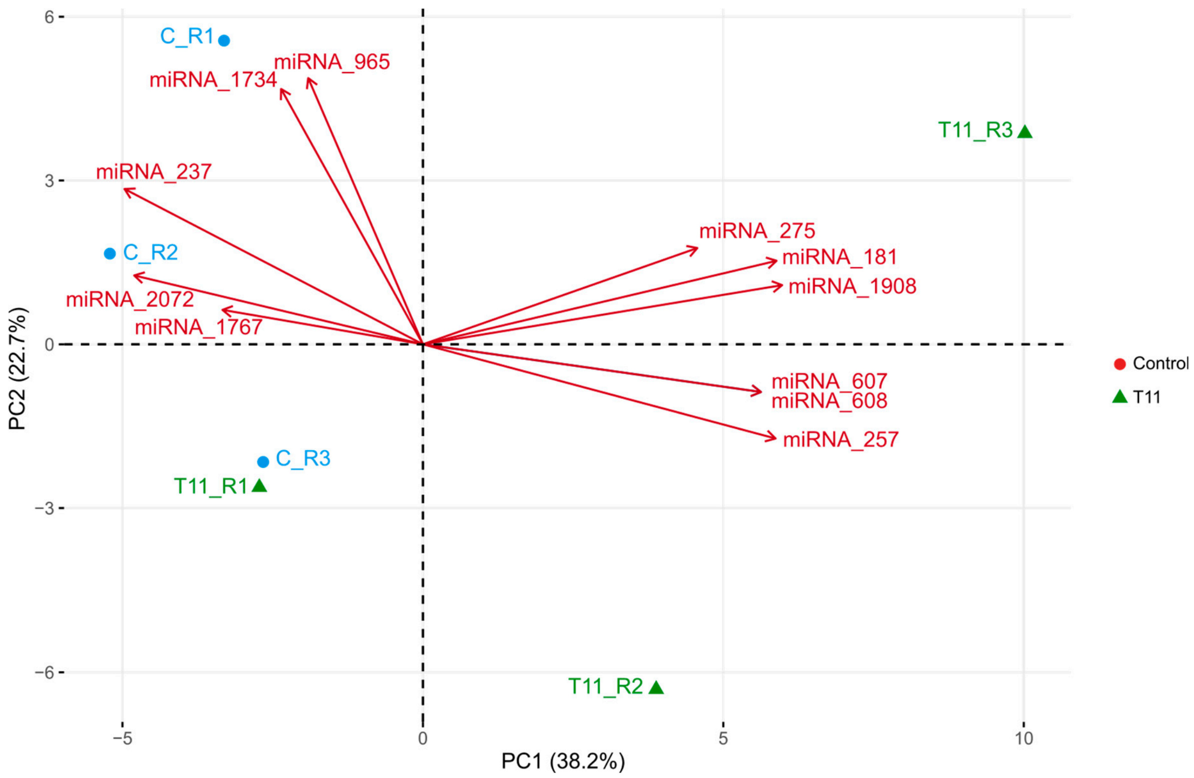

Figure S1. Principal Component Analysis (PCA) based on counts per million (CPM) values of the identified miRNA. The 10 DE miRNA selected for downstream analysis are shown as dark-red variables.
